# Pre-existing oscillatory activity as a condition for sub-harmonic entrainment of finely tuned gamma in Parkinson’s disease

**DOI:** 10.1016/j.brs.2024.02.017

**Published:** 2024-04-27

**Authors:** James J. Sermon, Philip A. Starr, Timothy Denison, Benoit Duchet

**Affiliations:** a MRC Brain Network Dynamics Unit, Nuffield Department of Clinical Neuroscience, University of Oxford, Oxford, United Kingdom; b Institute of Biomedical Engineering, Department of Engineering Sciences, University of Oxford, Oxford, United Kingdom; c Department of Neurological Surgery and Weill Institute for Neurosciences, University of California San Francisco, San Francisco, CA, USA

Dear Editor,

We have read with great interest the response by Scherer et al. to our recently published article [1], and would like to shed light on the key distinctions between the model presented by the authors, and our work. In both cases, the focus is on modelling finely tuned gamma (FTG) oscillations in patients with Parkinson’s disease (PD) receiving basal ganglia deep brain stimulation (DBS). However FTG is considered in different brain structures, specifically in the subthalamic nucleus (STN) in the letter by Scherer et al., and in the motor cortex in our work. More importantly, the letter by Scherer et al. sketches how FTG could be induced *de-novo* in the STN by DBS, whereas we predict stimulation parameters leading to the entrainment of *pre-existing* (i.e. medication induced) cortical FTG by DBS.

The spectral characteristics of FTG induced by DBS in the absence of dopaminergic medication are remarkably different from those of medication-induced FTG entrained by DBS. As reported by Wiest et al., DBS-induced FTG recorded in the STN in the off-medication state neither occurs at the half-harmonic of DBS frequency, nor shifts with changing stimulation frequency [2,3]. The persistence of DBS-induced FTG following stimulation cessation in the STN further illustrates that it is unlikely to be merely a consequence of entrainment by DBS. Furthermore, there is evidence that FTG can be induced without entrainment by DBS in the cortex in the off-medication state [4]. In contrast, medication-induced FTG can be entrained at exactly the half-harmonic of DBS frequency for a variety of stimulation parameters. This has been primarily demonstrated in the motor cortex [5,6,1,7], but was also observed in the STN [7]. Upon stimulation termination, FTG shifts back to its pre-stimulation frequency. Together, these findings suggest that half-harmonic entrainment may be more likely with a pre-existing oscillation.

To support this hypothesis, we investigate in a computational model the susceptibility to half-harmonic entrainment of a population of coupled Kuramoto oscillators as a function of the network coupling strength (see Supplementary Material for methodological details). As one of the simplest models that can describe populations of interconnected neurons, the Kuramoto model has frequently been used to represent oscillatory neural activity as well as the effect of DBS [8–10]. The level of coupling between oscillators controls the strength of the collective oscillatory activity emerging from the network. In the absence of stimulation, our oscillator population does not give rise to a significant collective oscillation when coupling is low (representing the off-medication case, see Fig. 1Ca), but generates a collective oscillation (parametrised to be at 75 Hz) when coupling is high (representing the medication-induced FTG case, see Fig. 1Cc). Providing DBS at 130 Hz increases population synchrony in the absence of a pre-existing collective oscillation (see Fig. 1A for low coupling strength). Yet, while the population peak frequency in the gamma range appears to shift towards 65 Hz, stimulation fails to significantly entrain the population at the half harmonic of stimulation (Fig. 1Bb and Fig. 1Cb). Conversely, when off-stimulation population synchrony is high enough (coupling strength>150), the pre-existing collective oscillation can robustly be entrained at the half-harmonic, see Fig. 1Bd and Fig. 1Cd. We note that stimulation amplitude should be high enough for half-harmonic entrainment to occur, but increasing stimulation amplitude past a certain threshold can lead to the loss of entrainment (Fig. 1B), as predicted by our previous modelling work and observed in data [1].

Contrary to the model by Scherer et al., the present model does not precisely elucidate the properties of off-medication DBS-induced FTG but offers complementary insight. Our analysis provides theoretical evidence that measurably entraining (at the half harmonic) a neural network not exhibiting a pre-existing collective oscillation is less likely than when a collective oscillation exists off stimulation (in the vicinity of the target frequency). It is worth highlighting that in real data, a weak collective oscillation amenable to entrainment may be masked by neural and measurement noise, or display non-stationary behaviour. Our framework may explain why medication-induced FTG can be entrained by DBS, and why there is no evidence that off-medication DBS-induced FTG can be, underscoring the critical role of dopaminergic medication in enabling FTG entrainment [7].

The clinical significance of motor-cortical but also subcortical medication-induced FTG entrainment at the half harmonic of DBS frequency was recently demonstrated [7]. In particular, Oehrn et al. reported that FTG entrained power can predict hyperkinetic states in PD patients at home. Additionally, FTG entrained power was identified as the optimal control signal for adaptive DBS aimed at reducing residual motor fluctuations, resulting in improved motor symptoms and quality of life at home compared to standard-of-care continuous DBS. Nevertheless, it remains to be investigated whether FTG induced by DBS in the off-medication state (in the absence of entrainment) can be a clinically significant signal.

## Supplementary Material

Supplementary material related to this article can be found online at https://doi.org/10.1016/j.brs.2024.02.017.

Supplementary material

## Figures and Tables

**Fig. 1:**
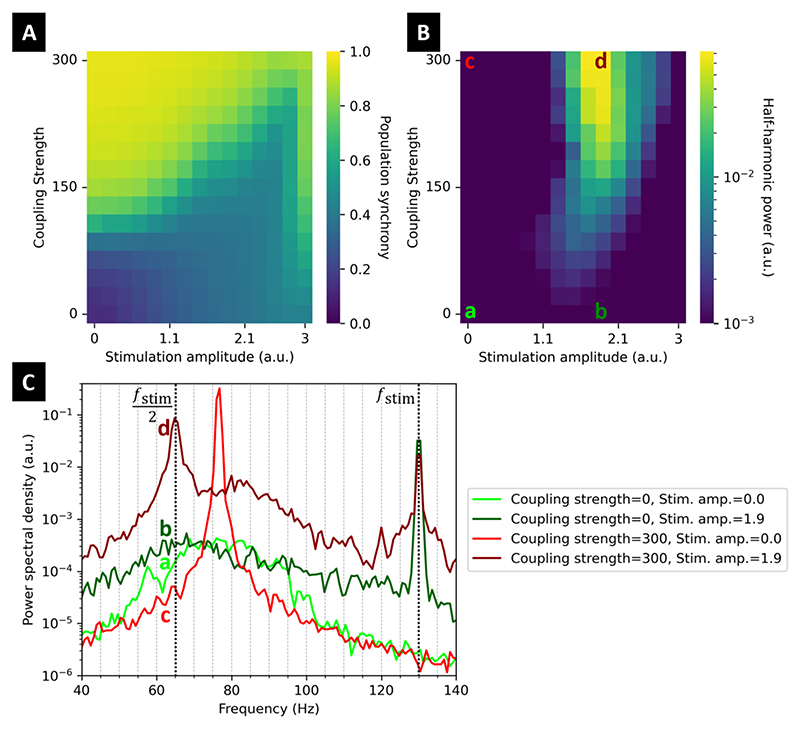
Pre-existing collective oscillatory activity is required for half-harmonic entrainment in a Kuramoto model. **A**: Population synchrony (time-averaged modulus of the network order parameter) reflects the strength of collective oscillatory activity in the network, and is shown here as a function of network coupling strength and stimulation amplitude. **B**: Peak power in the [*f*_stim_/2 − 2*Hz, f*_stim_/2 + 2*Hz*] frequency band, quantifying half-harmonic entrainment. Computed on the network output (real part of the order parameter). **C**: Power spectral density of the network output for low coupling (a and b), and high coupling (c and d), in the absence of stimulation (a and c), and with a stimulation amplitude leading to half-harmonic entrainment (b and d). Note the presence of half-harmonic entrainment for high coupling strength (d), and the absence of half-harmonic entrainment for low coupling strength (b). Annotations in colour show the correspondence with Panel C. Black dashed lines represent the stimulation frequency and its half harmonic. Stimulation is provided at *f*_stim_ = 130 Hz, and further methodological details can be found in Supplementary Material.

## Data Availability

No data were collected as part of this work.
