# Supplementary material for: Pre-existing oscillatory activity as a condition for sub-harmonic entrainment of finely tuned gamma in Parkinson’s disease
Source: Brain Stimul. Author manuscript; Available in PMC 2024 May 21. (PMC7615964; doi:10.1016/j.brs.2024.02.017)
Supplement: Supplementary material [file EMS196103-supplement-Supplementary_material.pdf]

Supplementary material

We use the Kuramoto model to investigate the susceptibility to half-harmonic entrainment of a neural population as a function of the network coupling strength and stimulation amplitude. We simulate $N = 50$ Kuramoto oscillators with noise and homogeneous coupling. The time evolution of the phase θ_k of the kth oscillator is described by the stochastic differential equation

$$\mathrm{d}\theta_k = \left[\omega_k + \frac{K}{N}\sum_{l=1}^{N}\sin(\theta_k - \theta_l) + I(t)Z(\theta_k)\right]\mathrm{d}t + \sigma \mathrm{d}W_k, \tag{1}$$

where ω_k is the intrinsic frequency of the kth oscillator, K is the coupling strength, $I(t)$ is the stimulation pulse train, Z is the oscillator phase response curve (PRC), $\sigma = 5$ is the model noise standard deviation, and W_k are independent Wiener processes. The order parameter of the network reads $\mathcal{Z}(t) = \Sigma_{k=1}^{N} e^{i\theta_k(t)}/N$, and the network synchrony is given by its modulus $\rho(t) = |\mathcal{Z}(t)|$. The network is simulated using a Euler-Maruyama scheme with time step $\Delta t = 10^{-4}$s.

The ω_k's are drawn from a normal distribution of mean 75Hz and standard deviation 7.5Hz. As an approximation of a type-II PRC, the PRC is taken to be $Z(\theta) = -\sin\theta$. The stimulation pulse train at time point t_i representing a simplified DBS pulse train is obtained as

$$I(t_i) = \begin{cases} \frac{A}{\Delta t} & \text{if } \left(t_i \mod \frac{1}{f_{\text{stim}}}\right) < \Delta t, \\ 0 & \text{otherwise,} \end{cases} \tag{2}$$

where $f_{\text{stim}} = 130$Hz is the stimulation frequency, and A is stimulation amplitude. Initial phases $\theta_k(0)$'s are drawn from a uniform distribution between 0 and 2π.

For each combination of 15 values of the coupling strength K and 15 values of the stimulation amplitude A, the network is simulated for 192 repeats of duration 10s each to obtain heatmaps representing population synchrony and peak half-harmonic power (Fig 1A and B). For each simulation, population synchrony is obtained as the temporal average of $\rho(t)$, and half-harmonic power is calculated as the peak power spectral density (PSD) in the band $[f_{\text{stim}}/2 - 2Hz, f_{\text{stim}}/2 + 2Hz]$. The PSD is computed on the real part of $\mathcal{Z}(t)$ with Welch's method using a Hann window and eight non-overlapping segments. Both population synchrony and half-harmonic power are then averaged across repeats. PSDs in Fig 1C are obtained as above based on 10s simulations.
